# Feline Cyst-like Lymphocytic Cholangiohepatitis in a Cat: First Case Report

**DOI:** 10.3390/ani12233278

**Published:** 2022-11-24

**Authors:** Sathidpak Nantasanti Assawarachan, Rungrueang Yodsheewan, Phudit Maneesaay, Kasem Rattanapinyopituk, Piyathip Chuchalermporn, Atijit Kongchun, Benjang Hakhen, Panpicha Sattasathuchana

**Affiliations:** 1 Department of Companion Animal Clinical Sciences, Faculty of Veterinary Medicine, Kasetsart University, 50 Ngamwongwan rd., Lat Yao, Jatujak, Bangkok 10900, Thailand; 2 Faculty of Veterinary Medicine, Kasetsart University Veterinary Teaching Hospital, Kasetsart University, 50 Ngamwongwan rd., Lat Yao, Jatujak, Bangkok 10900, Thailand; 3 Department of Pathology, Faculty of Veterinary Medicine, Kasetsart University, 50 Ngamwongwan rd., Lat Yao, Jatujak, Bangkok 10900, Thailand; 4 Department of Pathology, Faculty of Veterinary Science, Chulalongkorn University, 39 Henri Dunant rd., Pathumwan, Bangkok 10330, Thailand

**Keywords:** cat, cyst, cholangitis, cholangiohepatitis, liver

## Abstract

**Simple Summary:**

Cholangitis**/**cholangiohepatitis is the most common inflammatory disease of the biliary system in cats. A tissue biopsy is crucial for a definitive diagnosis. Lymphocytic cholangitis is one type of cholangitis and is identified through histological findings. Treatment requires a long-term immunosuppressive drug. This article is the first report of multiple cyst-like hepatic lesions with histological findings of lymphocyte infiltration in a cat’s liver.

**Abstract:**

A 5-year-old female neutered domestic short-haired cat presented with abdominal enlargement. An abdominal ultrasound revealed that large multiple hepatic cysts with irregular walls, hypoechoic fluid, and internal septations occupied most of the liver parenchyma. Serum liver enzymes, bilirubin, and bile acids concentrations were within normal limits. A fecal examination using simple floatation and formalin-ether sedimentation techniques was negative for liver fluke (*Platynosomum fastosum*), intestinal protozoa, and other helminth eggs. Praziquantel was prescribed for two distinct courses one month apart without obvious improvement of the hepatic cysts. An abdominal laparotomy and histopathological examination finally enabled diagnosis of cyst-like lymphocytic cholangiohepatitis of the liver tissue. Twelve weeks of oral prednisolone resulted in marked ultrasonographic improvement of the hepatic cysts. The liver parenchyma was heterogeneous and filled with multiple small anechoic cavities. Twenty-three months after ceasing the prednisolone, there was no recurrence of hepatic cysts.

## 1. Introduction

Feline cholangitis/cholangiohepatitis is an inflammatory condition of two conjunctive organs: the gall bladder and the liver [1,2]. The prevalence of feline cholangitis ranges from 25% to 50% across the world [2,3,4,5,6]. According to the World Small Animal Veterinary Association Standardization Committee, there are four forms of feline cholangitis—neutrophilic cholangitis, lymphocytic cholangitis, destructive cholangitis, and chronic cholangitis—associated with liver fluke infestation [7]. Differentiation of the four distinct forms requires a tissue biopsy [7]. Lymphocytic cholangitis is characterized by the presence of an aggregation of lymphocytes around the bile ducts. Cholangiohepatitis occurs when the inflammation extends to the hepatic parenchyma [7]. Cats with lymphocytic cholangitis/cholangiohepatitis commonly present with weakness, anorexia, nausea, vomiting, weight loss, ascites, and jaundice [5,8]. They may have increased hepatic enzyme activities, total bilirubin, and hypergammaglobulinemia [9,10]. Some cats may develop mild nonregenerative anemia or poorly regenerative anemia [9,10]. Ultrasonographic assessment of a cat with lymphocytic cholangitis/cholangiohepatitis may detect normal hepatic parenchyma or diffuse heterogeneous liver parenchyma and biliary system abnormalities, such as bile duct dilation, presence of gall bladder sludge, and gall bladder wall or bile duct wall thickening [2,5,11,12]. Lymphocytic cholangitis/cholangiohepatitis may be associated with an immune-mediated condition [8,13]. An immunosuppressive dosage of prednisolone is recommended for effective treatment to minimize hepatocellular damage and increase appetite [10].

Although lymphocytic cholangitis/cholangiohepatitis is commonly found, cystic lesions of the hepatic parenchyma have not been described in cats with lymphocytic cholangitis/cholangiohepatitis. The present article represents the first report of clinical signs and characteristics of cyst-like lesions on the hepatic parenchyma with lymphocytic cholangitis/cholangiohepatitis in a cat.

## 2. Case Description

A 5-year-old female neutered domestic short-haired cat (5.3 kg) was brought to the Kasetsart University Veterinary Teaching Hospital with a 1-month history of progressive abdominal enlargement. Informed consent was obtained from the cat’s owner. The cat had indoor and outdoor access and was fed a commercial, complete, and balanced cat diet. The cat was alert and had hyporexia. A complete physical examination was performed and vital parameters were within normal limits. The clinical examination revealed that the cat had pale mucous membranes and moderate abdominal distension. Hematology and blood chemistry testing was performed using an automated hematology analyzer (Sysmex XN-1000^TM^ Hematology Analyzer, Sysmex, Lincolnshire, IL, USA) and an automatic chemistry analyzer (IL Lab 650 chemistry system, Diamond Diagnostics, Holliston, MA, USA). A complete blood count indicated regenerative anemia with a hematocrit of 16.4% (reference interval (RI): 30–45%), an increased mean corpuscular volume of 60.07 fL (RI: 39–55 fL), absolute reticulocytosis of 225,000/µL (RI: 0–50,000/µL), and neutrophilia of 14,547 cells/µL (RI: 2500–12,500 cells/µL) (Table 1). A blood smear examination revealed anisocytosis, giant platelets, rouleaux formation, and toxic neutrophils. Serum creatinine, blood urea nitrogen, alanine aminotransferase (ALT), alkaline phosphatase (ALP), gamma-glutamyl transferase (GGT), bile acids, total protein, and albumin concentrations were within normal limits (Table 2). A high blood glucose concentration of 256 mg/dL (RI: 60–130 mg/dL) was found.

The results of a combination enzyme-linked immunosorbent assay (ELISA) test for the feline leukemia (FeLV) antigen and the feline immunodeficiency virus (FIV) antibody, using a commercially available FeLV–FIV rapid immunochromatography test assay (Witness^®^, Lyon Cedex, France), was negative. Doxycycline (10 mg/kg/day for 28 days; Siadocin^®^, Siam Pharmaceutical, Bangkok, Thailand) was prescribed orally for suspected *M. haemofelis* because the cat had regenerative anemia and lived in an area where the tick-borne parasite is endemic, although the blood smear results were negative for hemoparasites.

**Figure 1:**
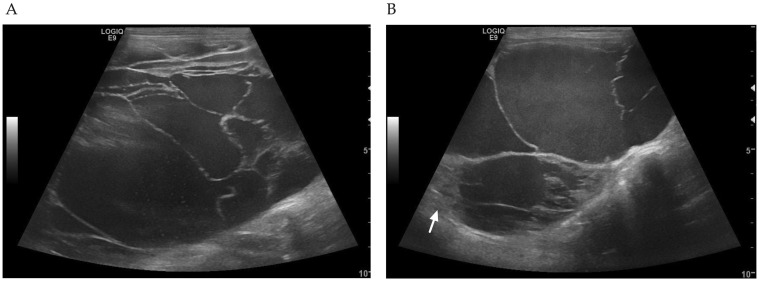
Ultrasonographic findings on the first visit. (**A**) Large hepatic cysts contained hypoechoic fluid and internal septations. (**B**) Hepatic cysts surrounded with irregular walls. There was a small area of mild heterogeneous liver parenchyma (arrow).

The abdominal ultrasound was performed by the Thai Board of Veterinary Surgeons, sub-specialty of veterinary diagnostic imaging, using a real-time scanner (LOGIQ E9, GE, Fairfield, CT, USA) with a 13 MHz broadband linear transducer. An abdominal ultrasound identified multiple large hepatic cysts occupying most of the liver (Figure 1). The cysts were characterized by thin and irregular walls with hypoechoic fluid and internal septations. There was only a small area of mild heterogeneous liver parenchyma. The other abdominal organs were unremarkable. Percutaneous ultrasound-guided needle aspiration of a hepatic cyst yielded a red and cloudy fluid. The fluid sample had a specific gravity of 1.035, a protein concentration of 5.7 g/dL, and a total nucleated cell count of 1910 cells/µL. Microscopic evaluation of the fluid sample revealed numerous erythrocytes, some macrophages, neutrophils, and lymphocytes. Fluid from the cysts was also submitted for a bacterial culture. No growth of bacteria was identified under aerobic or anaerobic conditions. Therefore, liver abscesses were ruled out. A fecal examination using simple floatation and formalin-ether sedimentation techniques was negative for liver fluke (*Platynosomum fastosum*), intestinal protozoa, and other helminth eggs. However, praziquantel (30 mg/kg/day; Opticide-FC^®^, Medicpharma Co., Ltd., Samut Sakhon, Thailand) was prescribed for 4 days.

After 4 weeks of doxycycline administration, the cat’s weight was 5.5 kg. The cat was alert and had a normal appetite. Moderate abdominal distension was still found during the physical examination. Blood tests showed signs of improvement of the anemia, with a hematocrit of 27.5% (RI: 30–45%) and a decreased mean corpuscular volume of 35.99 fL (RI: 39–55 fL).

One month after the first dose of praziquantel administration, the cat received the second dose of praziquantel in a similar fashion to the initial dose. However, there was no noticeable improvement of the abdominal enlargement and hepatic cysts as determined by a clinical examination and an ultrasonographic evaluation. Parasitic cysts due to *Platynosomum fastosum* infection were unlikely. Therefore, an exploratory laparotomy was performed. Anesthesia was induced with a slow IV infusion of propofol (8 mg/kg; Troypofol, Troikaa Pharmaceuticals Ltd., India) and then maintained with isoflurane (Attane™, Piramal Critical Care, Inc., Bethlehem, PA, USA). Intramuscularis injection of morphine sulphate (0.2 mg/kg; M&H Manufacturing Co., Ltd., Samutprakarn, Thailand) was used for analgesia. Multiple large hepatic cystic lesions occupied the liver parenchyma and could not be removed. There was a small area of non-cystic liver parenchyma. Therefore, incisional biopsies of the liver and hepatic cyst walls were collected for histopathology. The cat’s anesthetic recovery went well. Intravenous fluid therapy using 0.9% NaCl (General Hospital Products Public Co., Ltd., Pathum Thani, Thailand) and antibiotic therapy using amoxicillin-clavulanate intravenously (20 mg/kg q8h; Cavumox^®^, Siam Pharmaceutical Co., Ltd., Bangkok, Thailand) was continued for postoperative care. Tramadol (3 mg/kg q12h, subcutaneous; Tramada-100^®^, L.B.S. Laboratory Ltd. Part., Bangkok, Thailand) was administered for analgesia. The cat was bright with a good appetite and was discharged 72 h postoperatively, with the sutures removed 7 days later.

**Figure 2:**
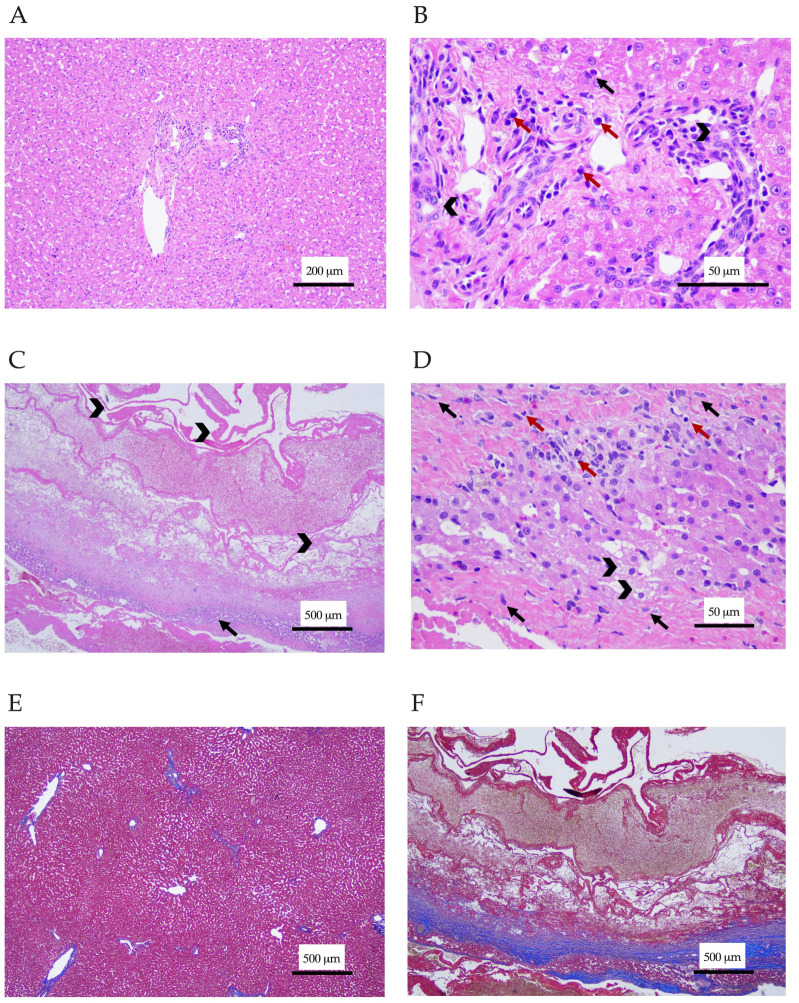
Histological section of the liver and cyst-like wall. (**A**,**B**) The liver section stained with hematoxylin-eosin showed infiltration of mononuclear inflammatory cells (red arrows) and neutrophils (black arrows) in periportal areas and the hepatic parenchyma. Bile duct epithelium cells are marked with arrowheads. Magnification ×100 (**A**) and ×400 (**B**). (**C**,**D**) The hepatic cyst-like wall section stained with hematoxylin-eosin. (**C**) Eosinophilic fibrinous proteinaceous materials rearranged as a cyst-like wall (arrowhead). The remaining hepatic cords (arrow) were infiltrated with a mix of inflammatory cells, including lymphocytes, plasma cells, macrophages, and some neutrophils. (**D**) Hepatic cords showed vacuolar degeneration of hepatocytes (arrowhead) and fibrosis (black arrow). Inflammatory cells (red arrow) were seen. Magnification ×40 (**C**) and ×400 (**D**). (**E**,**F**) The liver and cyst-like wall sections stained with Masson’s trichrome. (**E**) Normal collagen deposition around the portal areas and central veins presented in the liver section. (**F**) The cyst-like wall section had severe tissue fibrosis surrounding necrotic tissues. Magnification ×40 (**E**,**F**).

Three Thai board-certified pathologists analyzed the samples. Histopathology of the liver demonstrated lymphocytic cholangiohepatitis (Figure 2A,B). Mixed inflammatory cells including lymphocytes, plasma cells, macrophages, and a small number of neutrophils were seen in portal areas and had infiltrated the hepatic parenchyma. The hepatic cord was in a normal arrangement, but the hepatocytes exhibited cloudy swelling. Cyst wall sections revealed necrotizing hepatitis forming hepatic cyst-like lesions, which were composed of an abundance of eosinophilic fibrinous proteinaceous materials rearranging as a cyst-like wall without a lining of epithelial cells (Figure 2C,D). A small area of remaining hepatic cord showed the vacuolar degeneration of hepatocytes and infiltrated with mixed inflammatory cells, including neutrophils, lymphocytes, plasma cells, and macrophages. The necrotic lesion was surrounded by reactive fibroblastic cells, with thick fibrous connective tissue suggesting severe fibrosis. Tissue hemorrhage was also observed in the lesions. This finding confirmed the diagnosis of lymphocytic cholangiohepatitis. The Masson’s trichrome staining of liver section was performed to investigate the severity of fibrosis. The staining revealed normal collagen deposition around the portal areas and central veins, while the cyst-like wall section showed strongly positive staining, with the blue color indicating tissue fibrosis (Figure 2E,F). Prednisolone (1 mg/kg/day; Charoenbhaesaj Lab Co., Ltd., Bangkok, Thailand) was then prescribed. Two hepatoprotective agents, ursodeoxycholic acid (62.5 mg/d; Ursolin^®^, Berlin Pharmaceutical Industry Co., Ltd., Bangkok, Thailand) and silymarin (70 mg/day; Samarin^®^, Berlin Pharmaceutical Industry Co., Ltd., Bangkok, Thailand), were also administered.

**Figure 3:**
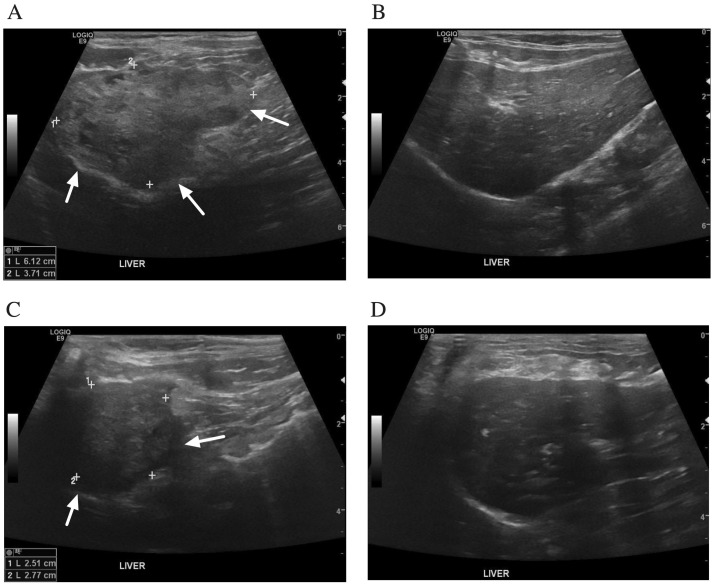
Ultrasonographic findings of the liver after treatment with prednisolone. (**A,B**) 12 weeks after prednisolone treatment. (**A**) The left lobe of liver showed a poorly defined mass (arrows) containing small irregular anechoic cavitations. (**B**) The right lobe of liver had a homogeneous parenchyma. There was no evidence of intrahepatic bile duct dilatation. (**C**,**D**) Twenty-three months after stopping prednisolone. (**C**) The left lobe of liver showed a heterogenous mass (arrows). (**D**) The right lobe of liver had a mild heterogenous parenchyma and round border. There was no evidence of common bile duct and intrahepatic bile duct dilatation.

After 12 weeks of prednisolone administration, the abdominal distension had disappeared. In addition, improvement of the hepatic cysts was detected by abdominal ultrasonography. The abdominal ultrasound demonstrated a heterogeneous liver parenchyma filled with irregular, small anechoic cavities on the left lobe of the liver (Figure 3A,B). The right lobe of the liver showed a homogeneous parenchyma. Thickening of the gall bladder wall and common bile duct dilatation were demonstrated. There was no evidence of intrahepatic bile duct dilatation. Prednisolone was then tapered down 25% every 2 weeks until reaching a physiological dose of 0.25 mg/kg/day. After 1 week of a physiological dose of prednisolone, prednisolone administration was stopped.

Twenty-three months after stopping prednisolone, the cat was doing well, was active, and had a good appetite. An ultrasonographic examination showed no recurrence or progression of the hepatic cysts (Figure 3C,D). The left lobe of the liver showed a decrease in the size of liver mass. The right lobe of the liver showed mild heterogenous parenchyma and a round border. The gall bladder wall was thickening. No evidence of common bile duct and intrahepatic bile duct dilatation was identified. Liver enzymes and liver function tests were within the reference intervals.

## 3. Discussion

The hepatic cyst-like lesions in this present case were diagnosed as lymphocytic cholangiohepatitis. In general, biochemical parameter abnormalities of cats with lymphocytic cholangitis/cholangiohepatitis are often revealed by an increase in one or more liver enzyme activities [1,3]. Interestingly, the hepatic cyst-like lesions of this cat were widespread and occupied most of the liver parenchyma, but liver enzymes and liver function tests were within normal limits. This finding may be explained by the 70–80% of hepatic reserve capacity [14]. The high blood glucose at the first visit may be explained by the non-sedated restraint procedure and chronic illness stage [15]. The secretion of catecholamines, cortisol, glucagon, and cytokines during stress contribute to gluconeogenesis [16]. The serum fructosamine concentration should be measured to exclude diabetic mellitus from stress-related hyperglycemia [15]. Nonetheless, serum fructosamine was not analyzed in the present case. The blood glucose concentration decreased below the renal threshold after the symptomatic treatment.

True hepatic cysts are fluid-filled cavities lined with epithelium; however, the pathogenesis is not well defined [17]. The cysts are often thin, have a well-defined wall and no internal echoes or septations, and lack the extent of hepatic parenchymal involvement [18,19,20]. These hepatic cysts may be accompanied by polycystic kidney disease; therefore, the kidney should also be evaluated [21,22,23]. Polycystic kidney disease is an autosomal dominant genetic-related disease in Persian cats [21,22,23,24]. In the cat described in this case report, polycystic kidney disease could be ruled out because the ultrasonography results for the kidneys were normal and the cat’s breed was domestic short-haired [24].

Clinical manifestation of liver fluke infestation can be hepatic cysts. Detection of liver fluke eggs from fecal examination may be missed because of the intermittent egg shedding and low number of eggs in feces [25]. Cytology of bile aspirate could increase the sensitivity of parasitic egg detection [26]. The prevalence of liver fluke was reported as 2.7–8.9% in cats residing in Thailand [26,27,28]. Therefore, praziquantel should be prescribed to all cats with hepatobiliary diseases in Thailand. Echinococcosis is another possible cause of hepatic cysts in cats [29]. To the authors’ knowledge, echinococcosis in cats has never been reported in Thailand.

On occasions, hepatic cysts become more complex lesions, characterized by irregular walls, septations, internal debris, or varying degrees of echogenic content. In these cases, neoplasia, hematoma, abscess, necrosis, inflammation, parasites, or toxins must be included in the differential diagnosis [30,31]. In our case, the hepatic cysts had complex internal features and involved all lobes of the liver. Liver abscess and hematoma had been ruled out by percutaneous aspiration of cystic content for cytology and a bacterial culture. Parasitic cysts due to liver flukes were also unlikely because the lesions did not improve after two distinct courses of praziquantel treatment. Therefore, a liver histology was performed for a final diagnosis, which was lymphocytic cholangiohepatitis.

Lymphocytic cholangitis/cholangiohepatitis in cats is characterized as the infiltration of small lymphocytes in the portal areas, with varying degrees of fibrosis and biliary proliferation. The pathogenesis of lymphocytic cholangitis/cholangiohepatitis was proposed to be involved with an autoimmune mechanism and bacterial infection [7,32]. Ultrasonographic appearances of liver parenchyma in cats with lymphocytic cholangitis/cholangiohepatitis may be reported to be normal, hypoechoic, or hyperechoic [5,11,12]. Common sonographic findings of the biliary tract can be dilation of the bile ducts, gall bladder distension, increased gall bladder sludge, thickening of the gall bladder wall, thickening of the bile duct wall, or unremarkable findings [2,12].

To the authors’ knowledge, complex cyst-like lesions of the liver parenchyma of a cat with lymphocytic cholangitis/cholangiohepatitis have not been reported. The cyst-like lesions contained the areas of necrotizing hepatitis, tissue hemorrhage, and eosinophilic fibrinous proteinaceous materials. The cause of hepatic cyst-like lesions in this case remains unclear. The hepatic cyst might be the primary cause. The gradual distention of the cysts might induce biliary obstruction, which promotes bacterial infection followed by cholangitis [33,34,35,36]. On the other hand, lymphocytic cholangiohepatitis may contribute to severe necrosis of the liver and, in the end, form cyst-like structures. This hypothesis may support by the presence of thick fibrous connective tissues surrounding necrotic lesions, which was manifested by the Masson’s trichrome staining of cyst-like wall section.

Treatment with anti-inflammatory dosages of prednisolone clearly improved the hepatic cyst-like lesions in this study, possibly because the cat was diagnosed early and showed normal liver function. We suggest histological analysis should be performed in cats with complex hepatic cysts that involve large parts of the hepatic parenchyma.

## 4. Conclusions

We have demonstrated the first case of feline lymphocytic cholangiohepatitis that presented with cyst-like structures in the liver. Histopathological analysis is important for diagnosis and leads to successful treatment with prednisolone.

## Figures and Tables

**Table 1:** Hematological profile at first visit, 4 weeks after doxycycline treatment, before prednisolone treatment, and 12 weeks after prednisolone treatment.

Hematological Parameters	Reference	Unit	First Visit	4 Weeks after Doxycycline Treatment	Before Prednisolone Treatment	12 Weeks after Prednisolone Treatment
Hemoglobin	10–15	g/dL	5.2	9.4	9.2	10.6
Packed red cells	30–45	%	16.4	27.5	29.1	31.1
Red blood cells	5–10	×10^6^ cells/µL	2.73	7.64	6.01	6.6
Mean corpuscular volume	39–55	fL	60.07	35.99	48.42	47.12
Mean corpuscular hemoglobin concentration	30–36	g/dL	31.71	34.18	31.62	34.08
Total white blood cells	5500–19,000	cells/µL	18,650	7410	8270	10,370
Segmented neutrophils	2500–12,500	cells/µL	14,547	5112.9	4854.5	6740.5
Lymphocytes	1500–7000	cells/µL	2797.5	1778.4	2299	3111
Monocytes	0–850	cells/µL	746	0	636.8	0
Eosinophils	0–750	cells/µL	559.5	518.7	479.7	518.5
Basophils	Rare	cells/µL	0	0	0	0
Absolute reticulocytes	0–50,000	/µL	225,000	9932	N/A	N/A
Platelets	300,000–800,000	cells/µL	471	146	52	229
Protein (refract)	6–7.5	g/dL	7.6	7.8	8	7.4
Blood morphology			Anisocytosis, rouleaux formation, giant platelets	Microcytes, giant platelets	Anisocytosis, giant platelets	Rouleaux formation, giant platelets

**Table 2:** Blood chemistry profile at first visit, 4 weeks after doxycycline treatment, before prednisolone treatment, and 12 weeks after prednisolone treatment.

Chemistry Parameters	Reference	Unit	First Visit	4 Weeks after Doxycycline Treatment	Before Prednisolone Treatment	12 Weeks after Prednisolone Treatment
Blood urea nitrogen	15–34	mg/dL	30	28	-	22
Creatinine	1.1–2.2	mg/dL	1.56	1.36	1.69	1.64
Glucose	60–130	mg/dL	256	157	-	180
Alanine aminotransferase	28–76	U/L	46	63	49	61
Alkaline phosphatase	0–62	U/L	22	-	-	33
Gamma-glutamyl transferase	0–4	U/L	1	-	-	1
Preprandial bile acids	0–6.9	µmol/L	5	-	-	-
Total bilirubin	0.1–0.5	mg/dL	0.1	-	-	0.1
Total protein	5.8–7.8	g/dL	6.7	7.4	8.3	7.4
Albumin	2.6–4.2	g/dL	3.1	3.0	3.4	3.8
Globulin	2.9–7.7	g/dL	3.6	4.4	4.9	3.6
Potassium	3.0–4.8	mEq/L	4.38	-	-	-

## Data Availability

All study data are presented in the article.
